# Validation and subcellular localization of previously predicted type III secreted effector proteins in *Chlamydia trachomatis*

**DOI:** 10.1128/spectrum.01616-25

**Published:** 2025-10-24

**Authors:** Paige N. McCaslin, Shelby E. Andersen, Parker Smith, Mary M. Weber, Robert Faris

**Affiliations:** 1 Department of Microbiology and Immunology, University of Iowa Carver College of Medicine 12243 , Iowa City, Iowa, USA; University of Nebraska Medical Center, Omaha, Nebraska, USA

**Keywords:** *Chlamydia trachomatis*, T3SS effector

## Abstract

The obligate intracellular bacterium *Chlamydia trachomatis* (*C.t*.) is the leading cause of bacterial sexually transmitted infections and infectious blindness worldwide. *C.t*. replicates within a specialized membrane-bound compartment known as the inclusion and employs a type III secretion system (T3SS) to deliver effector proteins into the host cell. These effectors reprogram host cellular processes to create an environment conducive to bacterial replication. Until recently, *C.t*. was genetically intractable, limiting efforts to identify effector proteins secreted during infection. As a result, early studies relied on heterologous expression in surrogate T3SS-component bacteria such as *Yersinia pseudotuberculosis*, *Shigella flexneri*, or *Salmonella enterica* serovar Typhimurium to assess secretion potential. With the advent of genetic tools for use in *C.t.*, we are now able to directly assess whether previously identified effectors are translocated during *C.t*. infection. Using three complementary approaches—the adenylate cyclase assay, β-lactamase assay, and glycogen synthase kinase assay—we identified 11 secreted effectors and 10 potentially secreted effectors. Of these 21 secreted factors, 10 displayed unique localization patterns when ectopically expressed in mammalian cells. Together, these findings define a core set of secreted effectors *C.t*. effectors that warrant further functional characterization.

**IMPORTANCE:**

This study systematically identifies and validates *Chlamydia trachomatis* type III secreted effectors, confirming secretion for 11 proteins and identifying 12 additional candidates. Using multiple complementary translocation assays, we directly assess secretion in the native bacterial context, revealing key effectors that target specific host compartments. Notably, several effectors localize to the nucleus or cytoplasm, suggesting distinct roles in host cell manipulation. Our findings refine the known effector repertoire and provide critical insights into how *C. trachomatis* remodels host organelles to sustain infection. By emphasizing experimentally verified secretion rather than surrogate systems, this work strengthens the foundation for future mechanistic studies and therapeutic targeting.

## INTRODUCTION

*Chlamydia trachomatis* (*C.t*.) is an obligate intracellular pathogen that causes the most prevalent bacterial sexually transmitted infection and is the leading cause of infectious blindness worldwide (1). In the United States alone, over 1.7 million chlamydial infections are reported annually, with an estimated 131 million cases occurring globally each year (2, 3). Urogenital infections with *C.t*. are often asymptomatic; however, untreated infections can ascend the genital tract and result in serious complications, including pelvic inflammatory disease, ectopic pregnancy, and infertility (2). Notably, infection does not confer lasting immunity, leading to high rates of reinfection and sustained transmission (4). These factors underscore the significant public health burden posed by *C.t*. and highlight the need to better understand its pathogenic mechanisms.

To replicate and cause disease, *C.t*. employs a unique biphasic developmental cycle, alternating between the infectious, elementary body (EB) and the replicative reticulate body (RB) (5). Upon contact with a host cell, pre-synthesized type III secretion system (T3SS) effector proteins are injected to induce cytoskeletal rearrangements and facilitate bacterial entry via endocytosis (6–13). After internalization, the EB is enclosed within a host-derived membrane-bound compartment known as the inclusion, which evades endolysosomal fusion and traffics along microtubules to the peri-Golgi region (14, 15). Within this protected niche, the EB differentiates into the RB and undergoes multiple rounds of replication before asynchronously converting back to the EB ~ 24–72 h post-infection (hpi) (16–18). At the end of the developmental cycle, the bacteria exit the host cell either via lytic cell rupture or by extrusion, a process in which the inclusion is released from the host cell while preserving host cell integrity (19).

From within the inclusion, *C.t*. extensively remodels the host cell environment to support its survival and replication. The pathogen manipulates a variety of host organelles and signaling pathways to acquire nutrients, evade immune responses, and maintain a hospitable intracellular niche (20, 21). Central to these interactions is the T3SS, which *C.t*. uses to translocate effector proteins directly into the host cell. Despite its small genome (1 Mb), *C.t*. is predicted to secrete 10%–15% of its proteome via the T3SS (20, 21), with these effectors broadly categorized as either inclusion membrane proteins (Incs) or conventional T3SS (cT3SS) effectors.

Inc proteins have one or two bilobed hydrophobic domains (~40 amino acids) that facilitate their insertion into the inclusion membrane, anchoring the protein such that its N-termini and C-termini are oriented into the host cell cytosol (22). *C.t*. is predicted to possess 58 Inc proteins, but only 38 have been experimentally confirmed to localize to the inclusion membrane (23–26), where they mediate crucial interactions with the host cell machinery (24, 27–33). In contrast, cT3SS effector proteins are secreted into the host cell cytosol and likely contribute to chlamydial infection at sites distant from the inclusion.

Identifying and characterizing cT3SS effectors remain challenging, as these proteins typically lack recognizable functional domains indicative of effector function, and they do not share homology with proteins of known function(s). Until recently, *Chlamydia* spp. were recalcitrant to genetic manipulation, necessitating the use of a surrogate system to identify secreted effectors. To date, more than 40 cT3SS effector proteins have been shown to be secreted using *Yersinia pseudotuberculosis*, *Shigella flexneri*, or *Salmonella enterica* serovar Typhimurium as surrogate hosts (34–36). While still challenging, advances in *Chlamydia* genetics, including the ability to transform *C.t*. with a stably maintained plasmid that enables inducible expression of epitope-tagged proteins, and the adoption of multiple reporter constructs have allowed for validation of effector protein secretion during infection (37–39). Significantly, several recent studies have confirmed that while useful, secretion by a surrogate organism does not necessarily correlate with secretion in the native organism (37, 40), necessitating validation of secretion by *C.t*.

In this study, we re-evaluated a panel of 36 candidate *C.t*. T3SS effector proteins that were previously screened in a surrogate bacterial system. By employing three complementary translocation assays, we sought to validate whether these candidates are secreted during active infection in the native host. Our systematic analysis confirmed 11 proteins as secreted and identified 10 additional proteins as possibly secreted. We further assessed the subcellular localization of these proteins by ectopic expression in mammalian cells and found that several effectors exhibit distinct localization patterns, suggesting interactions with specific host compartments. Collectively, this work expands the list of confirmed *C.t*. cT3SS effectors, underscores the importance of validating secretion in the native organism, and provides a valuable resource for future mechanistic studies into effector function.

## MATERIALS AND METHODS

### Bacterial and cell culture

*C.t*. serovar L2 (LGV 434/Bu) was propagated in HeLa 229 cells (American Type Culture Collection), and EBs were purified using a gastrografin density gradient as previously described (41). HeLa cells were grown in RPMI 1640 medium (Thermo Fisher Scientific) supplemented with 10% fetal bovine serum (Gibco) at 37°C with 5% CO_2_.

### Plasmid construction

To assess secretion of candidate cT3SS effectors, chlamydial genes were PCR amplified from L2/434/Bu genomic DNA, and each *orf* was cloned into the NotI/KpnI site of pBomb4 CyaA, pBomb4 BlaM, and pBomb4 GSK-FLAG (37). For ectopic expression, secreted effectors were cloned into the KpnI/XhoI site of pcDNA3.1-GFP. The integrity of all constructs was verified by DNA sequencing at McLab. All primers used in this study are listed in Table S1.

### Transformation of *C.t.*

*C. trachomatis* serovar L2 (LGV 434/Bu) EBs were transformed using 5 µg plasmid DNA and 10 µL 5× transformation mix (50 mM Tris, pH 7.4, and 250 mM CaCl_2_) in a total volume of 50 µL as previously described (37). RPMI with 10% fetal bovine serum (FBS) was added to each transformation and applied to two wells of a six-well plate containing a confluent HeLa cell monolayer. At 18 hpi, the medium was replaced with RPMI with 10% FBS containing 0.3 µg/mL penicillin G. Infectious progenies were harvested every 48 h and used to infect fresh HeLa cell monolayers until viable inclusions were evident (~2–3 passages). Expression of individual fusion proteins was confirmed by western blotting.

### Adenylate cyclase secretion assay

HeLa cell monolayers were infected at an multiplicity of infection (MOI) of 5 with *C.t*. transformants, and expression of the effector-adenylate cyclase (CyaA) fusion protein was induced using anhydrotetracycline hydrochloride (aTc; 10 ng/mL) as previously described (37). Cyclic AMP (cAMP) production in host cells was quantified by enzyme-linked immunosorbent assay (ELISA) according to the manufacturer’s guidelines (Abcam). Effector secretion was determined by comparing the levels of cAMP in cells infected with *C.t*. pBomb4 CyaA (negative control vector) to those infected with the *C.t*. CyaA-effector fusion strains.

### β-Lactamase assay

HeLa cells (2 × 10^4^/well) were seeded into black, clear bottom 96-well plates (Greiner). Cell monolayers were infected at an MOI of 5, and effector-β-lactamase (BlaM) fusion protein expression was induced using 10 ng/mL aTc as previously described (37). At 24 hpi, cells were washed three times with 1× phosphate-buffered saline (PBS) and loaded with CCF4-AM using the alternative loading protocol as per the manufacturer’s instructions (Thermo Fisher Scientific). Samples were incubated in the dark for 1 h at room temperature and were then read on a Tecan Infinite M200 Pro plate reader. To quantify effector translocation, the background was subtracted, the ratio of 460 nm to 535 nm (blue:green) was determined, and expression relative to cells infected with *C.t*. pBomb4 BlaM (negative control vector) was calculated as previously described (42).

### Glycogen synthase kinase-FLAG immunoprecipitation

Confluent HeLa cell monolayers in a T175 flask were infected at an MOI of 5, and expression of the effector-glycogen synthase kinase (GSK)-FLAG fusion was induced using 10 ng/mL aTc as previously described (37). At 24 hpi, cells were washed with ice-cold 1× PBS and lysed in 800 µL eukaryotic lysis solution (ELS) (50 mM Tris-HCl, 150 mM sodium chloride, 1 mM ethylenediaminetetraacetic acid, and 1% Triton-X 100) containing Halt cocktail protease and phosphatase inhibitor (Thermo Fisher Scientific) along with 10 µM GSK-3 α and β inhibitor 1-(7-methoxyquinolin-4-yl)-3-[6-(trifluoromethyl)pyridin-2-yl]urea (Tocris) as previously described (37). Supernatants were applied to anti-FLAG magnetic beads (Pierce Thermo Fisher Scientific) for 1.5 h at 4°C. The beads were subsequently washed 5× in ELS without Triton-X 100, and the purified protein was eluted using 4× NuPAGE LDS Sample Buffer (Thermo Fisher Scientific). Samples were analyzed by western blotting.

### Western blotting

For verification of CyaA or BlaM effector-fusion protein expression, confluent HeLa cell monolayers in a six-well dish were infected at an MOI of 5. At 24 hpi, the samples were lysed in ELS with Halt cocktail protease inhibitor (Thermo Fisher Scientific). For CyaA and BlaM, lysates were resolved using 3%–8% Tris-Acetate protein gels with Tris-Acetate sodium dodecyl sulfate (SDS) running buffer. For GSK-FLAG IPs, samples were resolved using 4%–12% Bis-Tris protein gels with (3-(N-morpholino)propanesulfonic acid) (MOPS)-SDS running buffer. Proteins were transferred to a polyvinylidene difluoride (PVDF) membrane and probed using anti-CyaA (Santa Cruz), anti-BlaM (QED BioScience), anti-GSK-3β (Cell Signaling), or anti-phospho-GSK-3β (Cell Signaling) antibodies.

### Immunofluorescence microscopy

To determine the subcellular localization of secreted effector proteins, HeLa cells were transfected with pcDNA3.1-GFP plasmids using Lipofectamine LTX (Thermo Fisher Scientific). Cells were fixed with 4% formaldehyde 18 h post-transfection, and the nucleus was stained using DAPI (Invitrogen). Images were captured on a Nikon Ti2 immunofluorescent microscope.

### Statistics

Statistical analysis was performed using GraphPad Prism 10.1.1 software. One-way analyses of variance (ANOVAs) were used followed by Tukey’s or Dunnett’s multiple comparisons with *, *P* < 0.05; **, *P* < 0.01; ***, *P* < 0.001; and ****, *P* < 0.0001.

## RESULTS

### Identification and re-evaluation of putative *Chlamydia trachomatis* type III secreted effectors previously screened in surrogate bacterial hosts

Over 29 putative *C.t*. cT3SS effectors have been identified based on their T3SS-dependent secretion in genetically tractable surrogate bacterial hosts (34–36, 43, 44). While these heterologous systems have proven valuable for initial screening, they can yield false positives and false negatives due to differences in effector recognition and export between species (37, 40). To comprehensively evaluate secretion by *C.t*. itself, we first curated a list of candidate effectors by systematically reviewing the literature for proteins previously identified in surrogate host screens conducted prior to the development of chlamydial genetic tools (34, 35, 43, 44). Additionally, we included several proteins from prior studies that were reported as non-secreted due to technical issues such as incorrect product size during western blotting (CT016), low expression levels (CT309), or inconclusive results (CT330, CT338, CT386, CT504, and CT631) (44). As positive controls, we included the well-characterized effectors TmeA and CteG, which have been confirmed to be secreted by *C.t*. during infection (38, 39).

### Effector translocation into host cells detected using a calmodulin-activated CyaA reporter system

**Fig 1:**
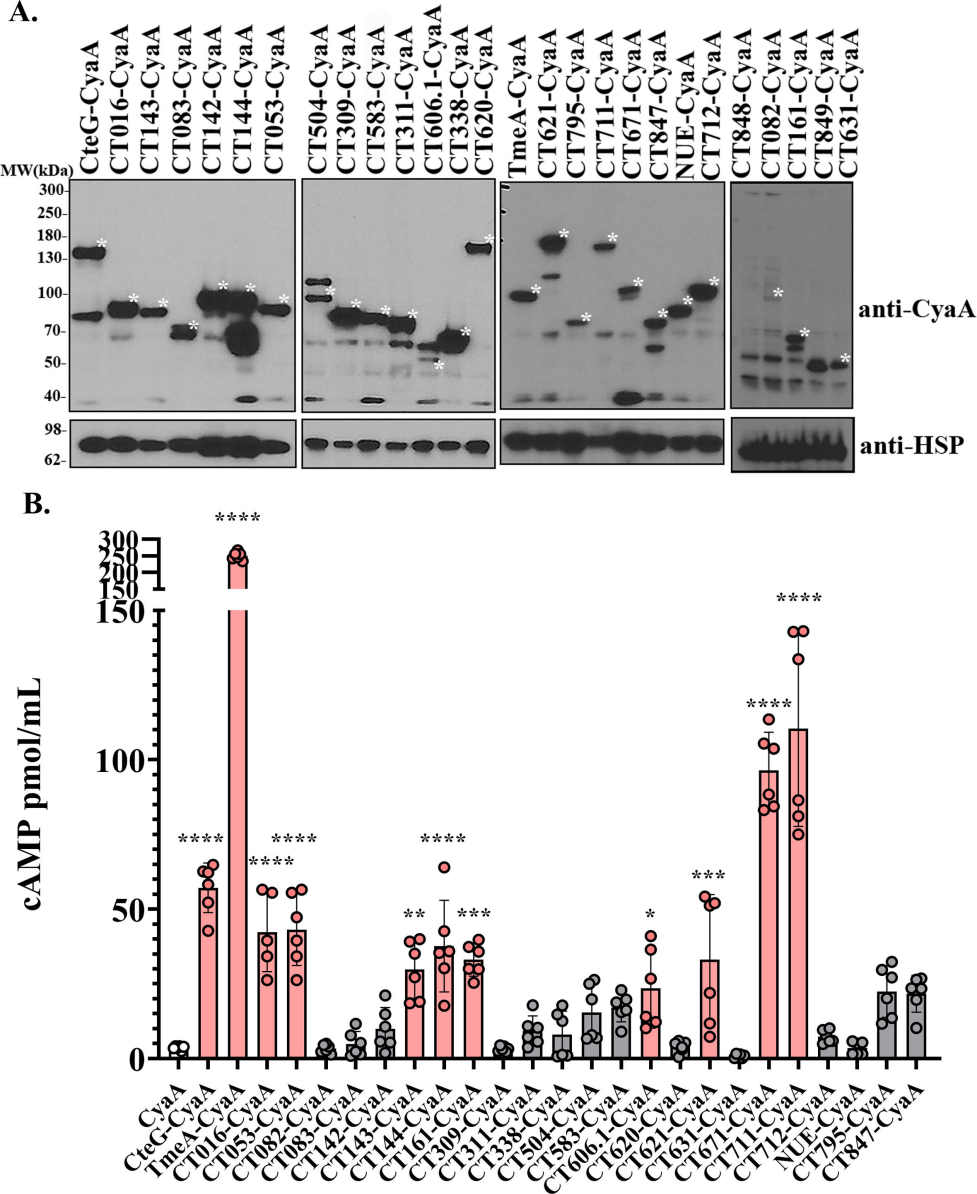
Detection of novel *C.t*. secreted effectors using the CyaA translocation assay. Candidate effector proteins were expressed as C-terminal fusions to the CyaA reporter and transformed into *C.t*. (**A**) HeLa cells were infected with the resulting *C.t*. transformants at an MOI of 5 for 24 h. Fusion protein expression was verified by western blotting using anti-CyaA antibodies, with *C.t*. heat shock protein (HSP) serving as a loading control. Asterisks indicate the observed molecular weights of the expressed CyaA fusion proteins. Predicted molecular weights for each fusion are as follows: CteG-CyaA: 113 kDa, CT016-CyaA: 71 kDa, CT143-CyaA: 76 kDa, CT083-CyaA: 63 kDa, CT142-CyaA: 76 kDa, CT144-CyaA: 76 kDa, CT053-CyaA: 62 kDa, CT504-CyaA: 77 kDa, CT309-CyaA: 77 kDa, CT583-CyaA: 66 kDa, CT311-CyaA: 71 kDa, CT606.1-CyaA: 53 kDa, CT338-CyaA: 63 kDa, CT620-CyaA: 138 kDa, TmeA-CyaA: 79 kDa, CT621-CyaA: 137 kDa, CT795-CyaA: 63 kDa, CT711-CyaA: 131 kDa, CT671-CyaA: 76 kDa, CT847-CyaA: 64 kDa, NUE-CyaA: 70 kDa, CT712-CyaA: 89 kDa, CT848-CyaA: 63 kDa, CT082-CyaA: 104 kDa, CT161-CyaA: 72 kDa, CT849-CyaA: 62 kDa, CT631-CyaA: 54 kDa. (**B**) Infected HeLa cells were assessed for cytosolic cAMP accumulation 24 h post-infection by ELISA. cAMP levels from cells infected with *C.t*. expressing each candidate fusion were compared to those infected with *C.t*. expressing CyaA alone. Samples with pink bars are positive for translocation. Error bars represent standard deviations, and statistical significance was determined using one-way ANOVA (****, *P* < 0.0001; ***, *P* < 0.001; **, *P* < 0.01; and *, *P* < 0.05). Data are from two independent experiments with three technical replicates per experiment.

To assess whether candidate cT3SS substrates are translocated into the host cytosol during *C.t*. infection, we employed a translocation assay based on the *Bordetella pertussis* calmodulin-dependent CyaA reporter. In this system, candidate effectors were expressed in *C.t*. as C-terminal fusions to CyaA. Upon secretion into the host cytosol, the CyaA domain becomes activated by host calmodulin, converting ATP to cAMP. Intracellular cAMP levels, quantified by ELISA, serve as a sensitive and quantitative readout of effector translocation. Using TmeA-CyaA and CteG-CyaA as positive controls, we observed significantly elevated cAMP levels in infected HeLa cells compared to those infected with *C.t*. expressing CyaA alone, validating the sensitivity of this assay (Fig. 1).

We applied this approach to 36 candidate effectors, detecting expression of 23 by western blot (Fig. 1A) and identifying 9 that were robustly secreted, as evidenced by significantly increased cAMP production: CT016, CT053, CT143, CT144, CT161, CT606.1, CT621, CT671, and CT711 (Fig. 1B). For several candidates, including CT082, CT083, CT142, CT309, CT311, CT338, CT504, CT583, CT620, CT631, CT712, CT737/NUE, CT795, and CT847, cAMP levels remained comparable to the CyaA-alone control, despite effector expression (Fig. 1A), suggesting these proteins are not secreted into the host cell or are below the detection threshold. Two effectors shown in Fig. 1A were excluded from Fig. 1B due to lack of detectable expression by western blot (CT848 and CT849). These results demonstrate that the CyaA translocation assay is a robust method for detecting secretion during *C.t*. infection and support the designation of multiple candidate proteins as *bona fide* secreted effectors (Fig. 1A; Table 1).

**TABLE 1:** Candidate cT3SS effectors evaluated for secretion in *C.t*. (ND, not determined)

D/UW-3/CX	L2/434/Bu	Reference	MW (kDa)	CyaA assay	BlaM assay	GSK assay	Final designation
CT016	CTL0271	(44)	26.7	Secreted	Not secreted	Secreted	Secreted
CT053	CTL0309	(44)	17.2	Secreted	Secreted	Secreted	Secreted
CT082	CTL0338	(43–45)	60	Not secreted	Not secreted	ND^*c*^	Not secreted
CT083	CTL0338A	(35)	18.3	Not secreted	ND^*b*^	ND^*c*^	Unable to conclude
CT142	CTL0397	(44, 46)	31.4	Not secreted	Not secreted	Secreted	Possibly secreted
CT143	CTL0398	(44, 46)	31.5	Secreted	Not secreted	Secreted	Secreted
CT144	CTL0399	(44, 46)	31.4	Secreted	Secreted	Secreted	Secreted
CT161	CTL0417	(44)	28.0	Secreted	Not secreted	Secreted	Secreted
CT163 (Lda2)	CTL0419	(47)	59.1	ND^*b*^	ND^*b*^	Not secreted	Unable to conclude
CT309	CTL0561	(44)	32.1	Not secreted	Not secreted	ND^*d*^	Not secreted
CT311	CTL0563	(48)	26.3	Not secreted	ND^*c*^	Secreted	Possibly secreted
CT330	CTL0584	(44)	10.0	ND^*b*^	ND^*b*^	ND^*b*^	Unable to conclude
CT338	CTL0592	(44)	17.8	Not secreted	Not secreted	ND^*d*^	Not secreted
CT386	CTL0642	(44)	33.1	ND^*c*^	ND^*a*^	Secreted	Possibly secreted
CT429	CTL0688	(44)	39.2	ND^*c*^	ND^*c*^	ND^*d*^	Unable to conclude
CT504	CTL0766	(44)	32.0	Not secreted	Not secreted	Secreted	Possibly secreted
CT550	CTL0812	(35)	15.8	ND^*a*^	ND^*c*^	ND^*b*^	Unable to conclude
CT583	CTL0846	(44)	30.7	ND^*c*^	Not secreted	Secreted	Possibly secreted
CT606.1	CTL0870	(35)	8.9	Secreted	ND^*c*^	ND^*c*^	Possibly secreted
CT610	CTL0874	(35)	26.8	ND^*b*^	Not secreted	ND^*c*^	Unable to conclude
CT620	CTL0884	(34)	93.2	Not secreted	Secreted	Secreted	Secreted
CT621	CTL0885	(34)	92.6	Secreted	ND^*b*^	Secreted	Secreted
CT622	CTL0886	(49)	21.7	ND^*a*^	Secreted	Secreted	Secreted
CT631	CTL0895	(44)	9.2	Not secreted	ND^*b*^	Secreted	Possibly secreted
CT652.1	CTL0021	(35)	6.6	ND^*c*^	Secreted	ND^*a*^	Possibly secreted
CT656	CTL0025	(44)	11.2	ND^*c*^	ND^*c*^	ND^*c*^	Unable to conclude
CT671	CTL0040	(35)	31.0	Secreted	Secreted	Secreted	Secreted
CT711	CTL0080	(34)	86.3	Secreted	Not secreted	Secreted	Secreted
CT712	CTL0081	(34)	44.1	Not secreted	Not secreted	Secreted	Possibly secreted
CT718	CTL0087	(35)	19.6	ND^*c*^	ND^*c*^	ND^*c*^	Unable to conclude
CT737 (NUE)	CTL0106	(50)	25.7	Not secreted	Not secreted	Not secreted	Not secreted
CT738	CTL0107	(35)	29.1	ND^*c*^	Secreted	Secreted	Secreted
CT795	CTL0164	(51)	17.9	Not secreted	Not secreted	ND^*b*^	Not secreted
CT847	CTL0219	(36)	18.9	Not secreted	ND^*b*^	ND^*b*^	Unable to conclude
CT848	CTL0220	(35)	18.3	ND^*c*^	ND^*b*^	Secreted	Possibly secreted
CT849	CTL0221	(44)	17.5	ND^*c*^	ND^*c*^	Not secreted	Unable to conclude

^*a*^ Unable to generate clone.

^*b*^ Unable to obtain chlamydial transformants.

^*c*^ Not expressed.

^*d*^ Skipped, determined designation from two assays already.

### Validation of candidate effector secretion using a BlaM-based translocation assay

To further assess candidate effector secretion, we employed a second translocation assay based on the BlaM reporter. BlaM was fused to the C-terminus of each candidate, and secretion into the host cytosol was evaluated using the fluorogenic substrate CCF4-AM. CCF4-AM contains two fluorescent moieties separated by a β-lactam ring; in its uncleaved state, fluorescence resonance energy transfer (FRET) between the dyes results in green fluorescence and emission at 535 nm. However, if BlaM is translocated into the host cytosol, it cleaves the β-lactam ring, disrupting FRET and shifting the fluorescence emission to blue (460 nm). The ratio of blue to green fluorescence (460:535 nm) serves as a quantitative readout of effector secretion. This approach has been successfully used to detect translocation of bacterial effectors in a variety of intracellular pathogens, including *C.t*. (37, 38, 42, 52, 53).

**Fig 2:**
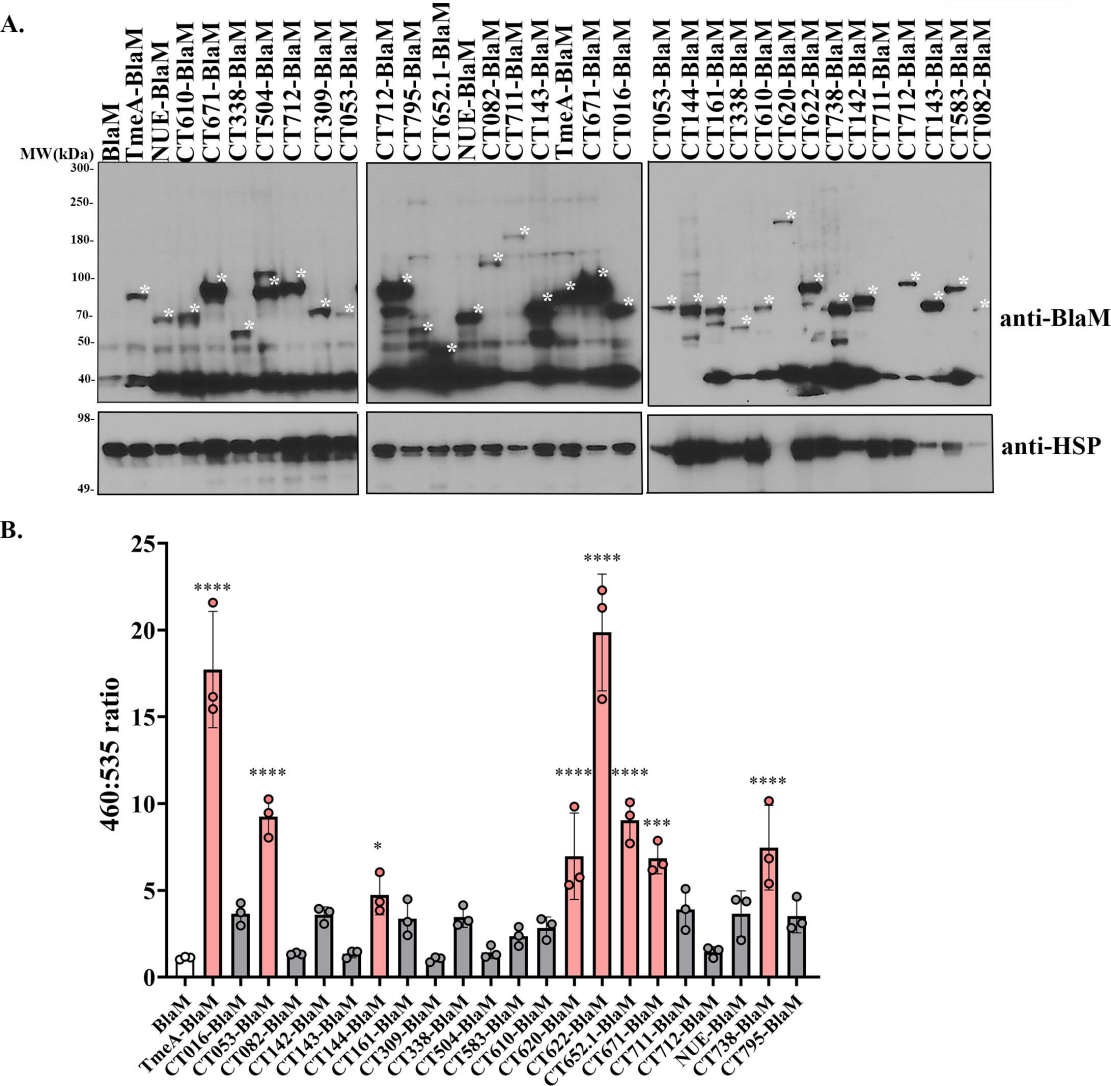
Detection of novel *C.t*. secreted effectors using the BlaM secretion assay. Candidate effector proteins were expressed as C-terminal fusions to the BlaM reporter and transformed into *C.t*. (**A**) HeLa cells were infected with the resulting *C.t*. transformants at an MOI of 5 for 24 h. Expression of each BlaM fusion protein was confirmed by western blotting using anti-BlaM antibodies, with *C.t*. heat shock protein (HSP) serving as a loading control. Asterisks indicate the observed molecular weights of each BlaM fusion protein. Predicted molecular weights are as follows: TmeA-BlaM: 64 kDa, NUE-BlaM: 55 kDa, CT610-BlaM: 56 kDa, CT671-BlaM: 61 kDa, CT338-BlaM: 48 kDa, CT504-BlaM: 62 kDa, CT712-BlaM: 74 kDa, CT309-BlaM: 62 kDa, CT053-BlaM: 47 kDa, CT795-BlaM: 48 kDa, CT652.1-BlaM: 37 kDa, CT082-BlaM: 89 kDa, CT711-BlaM: 116 kDa, CT143-BlaM: 61 kDa, CT016-BlaM: 56 kDa, CT144-BlaM: 61 kDa, CT161-BlaM: 57 kDa, CT620-BlaM: 123 kDa, CT622-BlaM: 99 kDa, CT738-BlaM: 59 kDa, CT142-BlaM: 61 kDa, CT143-BlaM: 61 kDa, CT583-BlaM: 60 kDa. Protein fusions repeated on multiple blots are NUE-BlaM, CT610-BlaM, CT671-BlaM, CT338-BlaM, CT712-BlaM, CT053-BlaM, CT082-BlaM, CT711-BlaM, CT143-BlaM. (**B**) Infected HeLa cells were loaded with CCF4-AM for 1 h, and fluorescence emission was monitored using a plate reader. Secretion into the host cell cytosol leads to cleavage of the β-lactam ring, which disrupts FRET and shifts fluorescence from 535 nm to 460 nm. The 460:535 nm ratio for each candidate was compared to *C.t*. expressing BlaM alone. Samples shown in pink are positive for translocation. Error bars represent standard deviations, and statistical significance was determined using one-way ANOVA (****, *P* < 0.0001; ***, *P* < 0.001; and *, *P* < 0.05). Data are graphed from one of three independent experiments with three technical replicates.

We validated the assay using TmeA-BlaM, which was previously shown to be secreted using this assay (38). We identified 21 BlaM fusion proteins that were expressed by western blot (Fig. 2A), all of which were evaluated in the BlaM secretion assay. Seven proteins—CT053, CT144, CT620, CT622, CT652.1, CT671, and CT738—were secreted by *C.t*. (Fig. 2B; Table 1). Despite the expression of the BlaM-effector fusion (Fig. 2A), we were unable to detect secretion of CT016, CT082, CT142, CT143, CT161, CT309, CT338, CT504, CT583, CT610, CT711, CT712, NUE, and CT795. Collectively, these data reinforce the utility of the BlaM reporter as a complementary method for validating effector secretion into the host cytosol during infection.

### Detection of cytosolic effector translocation using the GSK assay

Lastly, we employed a third translocation assay using the GSK epitope tag to evaluate candidate effector secretion. The GSK tag is a small, 13-residue tag that is specifically phosphorylated in the host cell cytosol by host kinases (54). This assay offers several advantages, including minimal disruption of effector structure due to the small size of the tag and ease of detection by western blotting with anti-GSK (expression) and anti-phospho-GSK (secretion) antibodies. To enhance signal detection and reduce background caused by nonspecific antibody binding, we appended a FLAG tag to the GSK-tagged effectors, enabling enrichment of the fusion proteins by immunoprecipitation prior to western blotting.

**Fig 3:**
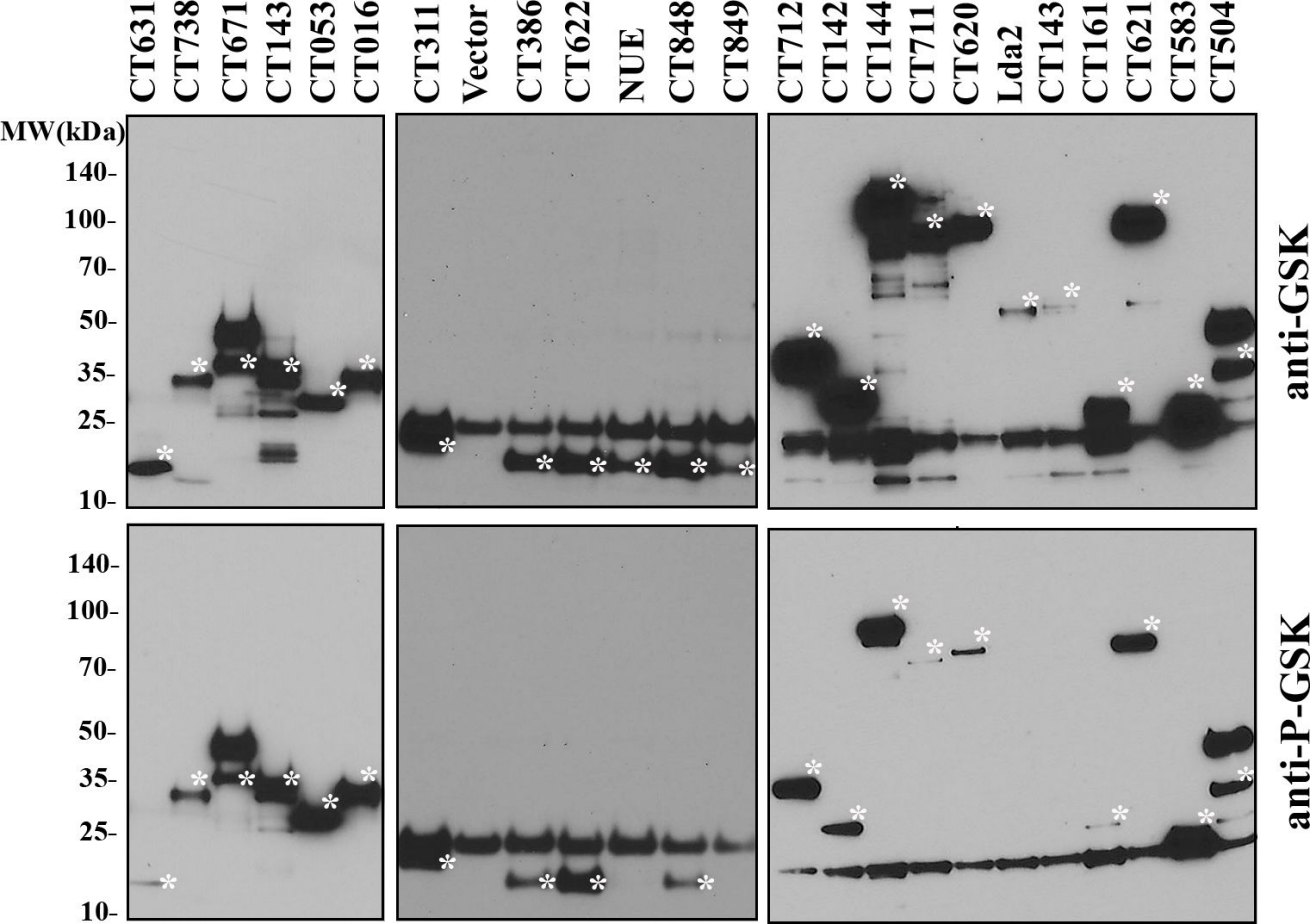
Detection of *C.t*. secreted effectors using the GSK-FLAG secretion assay. Candidate effector proteins were expressed as C-terminal fusions to a GSK-FLAG tag and transformed into *C.t*.. HeLa cells were infected with each strain at an MOI of 5, and at 24 hpi. Cell lysates were collected and subjected to immunoprecipitation using anti-FLAG magnetic beads. Samples were resolved by SDS-PAGE and analyzed by western blotting with anti-GSK antibodies to assess expression and anti-phospho-GSK (P-GSK) antibodies to detect secretion into the host cytosol. An asterisk indicates the predicted molecular weight of each GSK-FLAG fusion protein. Predicted molecular weights are as follows: CT631-GSK-FLAG: 13 kDa, CT738-GSK-FLAG: 33 kDa, CT671-GSK-FLAG: 35 kDa, CT143-GSK-FLAG: 35 kDa, CT053-GSK-FLAG: 21 kDa, CT016-GSK-FLAG: 30 kDa, CT311-GSK-FLAG: 30 kDa, CT386-GSK-FLAG: 37 kDa, CT622-GSK-FLAG: 73 kDa, NUE-GSK-FLAG: 29 kDa, CT848-GSK-FLAG: 22 kDa, CT849-GSK-FLAG: 21 kDa, CT712-GSK-FLAG: 49 kDa, CT142-GSK-FLAG: 35 kDa, CT144-GSK-FLAG: 35 kDa, CT711-GSK-FLAG: 90 kDa, CT620-GSK-FLAG: 97 kDa, Lda2-GSK-FLAG: 63 kDa, CT161-GSK-FLAG: 31 kDa, CT621-GSK-FLAG: 96 kDa, CT583-GSK-FLAG: 34 kDa, CT504-GSK-FLAG: 36 kDa. CT143 is present on two of the blots, where in the left blot it is highly expressed and secreted, and on the right blot it is not secreted, but lowly expressed. The following fusions were determined to be secreted based on the detection of phosphorylated GSK: CT016, CT053, CT142, CT143, CT144, CT161, CT311, CT386, CT504, CT583, CT620, CT621, CT622, CT631, CT671, CT711, CT712, CT738, and CT848. Data shown are representative of three independent experiments.

Using this approach, we identified 22 GSK fusion proteins as expressed and 19 proteins—CT016, CT053, CT142, CT143, CT144, CT161, CT311, CT386, CT504, CT583, CT620, CT621, CT622, CT631, CT671, CT711, CT712, CT738, and CT848—as secreted (Fig. 3; Table 1). We were unable to detect the secretion of NUE, CT849, and Lda2. The GSK assay appeared to be the most sensitive of the three translocation methods, likely due to the small size of the tag enabling more efficient translocation through the T3SS.

To classify secretion status across all candidates, we required that an effector be positive in at least two assays to be designated as “secreted,” positive in only one assay to be considered “possibly secreted,” and negative in at least two assays with no positives in any assay to be “not secreted.” Applying this framework, we validated 11 proteins as secreted effectors and identified an additional 10 as possibly secreted among the 36 candidates tested (Table 1).

### A subset of cT3SS effectors targets specific host compartments

**Fig 4:**
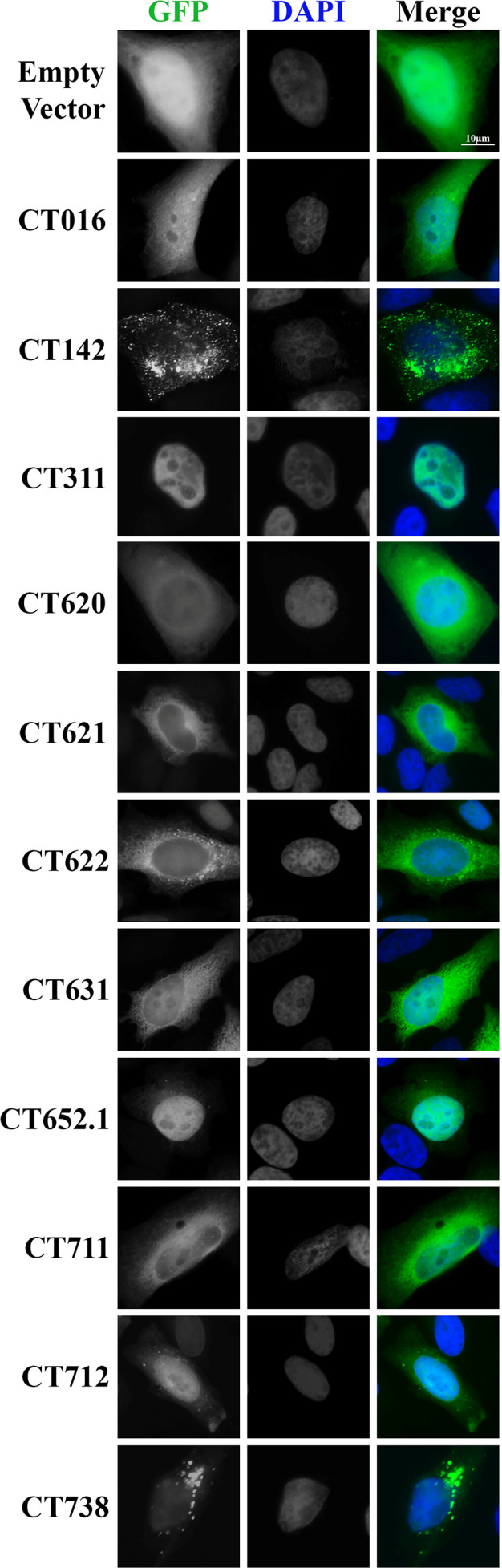
Subcellular localization of secreted and possible secreted *C.t*. effectors. Effector proteins identified as secreted or possibly secreted were fused to GFP and transiently transfected into HeLa cells to assess subcellular localization. At 18 h post-transfection, cells were fixed in 4% formaldehyde and stained with DAPI to visualize nuclei. Samples were imaged by immunofluorescence microscopy, and localization patterns were compared to those of GFP alone. Data are representative of three independent experiments.

Upon translocation into host cells, many bacterial effector proteins localize to specific subcellular compartments where they modulate host cell functions (52, 53, 55, 56). To determine whether these *C.t*. secreted effectors localize to defined cellular compartments, we examined the subcellular distribution of the 11 secreted and 10 possibly secreted effectors. Each effector was ectopically expressed in HeLa cells as a GFP-fusion, and localization was assessed by immunofluorescence microscopy. Of the 21 GFP-tagged effector proteins, 10 displayed localization patterns distinct from GFP alone (Fig. 4). CT311 and CT652.1 localized to the nucleus. CT620, CT621, CT622, CT631, CT711, and CT712 were diffusely cytosolic and excluded from the nucleus. In contrast, CT142 and CT738 showed a punctate cytoplasmic distribution, suggesting a potential association with vesicular or aggregated structures. CT016 displayed localization similar to GFP alone and was included as an example of no pattern. The other 10 secreted or possibly secreted effectors, not shown in Fig. 4, similarly had no discernible localization pattern. These findings indicate that a subset of *C.t*. effectors target specific host compartments, supporting a model in which effector localization contributes to their function during infection.

## DISCUSSION

 Prior to the advent of genetic tools for use in *C.t.*, over 30 putative cT3SS effectors had been shown to be secreted using genetically tractable surrogate hosts, including *Yersinia*, *Shigella*, or *Salmonella*. While useful, surrogate systems have been shown to result in false positives and negatives. In this study, we sought to systematically validate whether these candidate effectors are secreted during active *C.t*. infection using three complementary assays adapted for use in the native organism. By evaluating secretion within the context of infection, our approach accounts for pathogen-specific factors, offering a more accurate assessment of effector translocation. Our results underscore the importance of verifying surrogate system findings in the native context and expand the repertoire of *C.t*. effectors confirmed to be secreted during infection.

Of the 36 effectors we sought to test for secretion in *C.t*., 21 were found to be secreted in at least one assay. Due to challenges generating clones, obtaining chlamydial transformants, and/or lack of expression during *C.t*. infection, we were unable to draw conclusions for 10 of these candidates. Of note, CT082, NUE, and CT795 were not found to be secreted in our assays (Table 1). NUE was previously shown to be secreted using *Shigella flexneri* as a surrogate host, and mechanistic characterization revealed that it localizes to the nucleus and interacts with chromatin (50). It is possible that the timing of our assays may have limited detection of NUE secretion. Although NUE has been reported as a late secreted effector (50), all of our secretion assays were performed at 24 hpi, which may not capture the full temporal range of effector secretion. Additionally, since expression in our system is driven by a Tet-inducible promoter, the timing of transcription and subsequent secretion may not fully recapitulate native expression dynamics. A more detailed time-course analysis may be necessary to determine whether NUE is secreted at later stages of infection or under specific conditions not tested in our current study.

CT082 was previously identified as a secreted effector using *Yersinia enterocolitica* and *Y. pseudotuberculosis* surrogate systems, yet we did not detect secretion in *C.t*. infected cells in this study. In line with our results, previous studies utilizing a monospecific polyclonal antibody were unable to detect CT082 in the cytoplasm of *C.t*. infected cells and instead associated it with the bacterial cell envelope, suggesting that CT082 may not be a T3SS effector (40). Prior studies found that CT082 is first detected at 26 hpi (44). In our study, expression of CT082 was driven by an aTc-inducible promoter and secretion was assessed at 24 hpi, potentially before the native expression window and in the absence of other temporally regulated factors required for secretion.

CT795 was previously detected in the cytosol of infected host cells and shown to be secreted in *Escherichia coli*. However, secretion was blocked only when C16, an inhibitor targeting signal peptidase I - required for Sec system secretion, was added to *C.t*. infected cells. When the type III secretion system was blocked by the addition of a C1 compound, secretion of CT795 remained unaffected (51). The C1 compound has previously been shown to inhibit T3SS secretion in multiple organisms, including *C.t*. (57). Therefore, CT795 secretion likely depends on the Sec system and not the T3SS apparatus. It remains unknown how CT795 could be translocated from the periplasm, across the inclusion membrane, into the cytosol of the host cell (51), which could explain why we were unable to detect secretion in our assays.

Here, we evaluated the subcellular localization of all secreted and possibly secreted effectors. Of these, CT311 and CT652.1 localized to the nucleus. CT311 has previously been shown to contain a nuclear localization signal and localizes to the host cell nucleus during infection (48), consistent with our findings of nuclear localization during ectopic expression in uninfected cells. These results suggest that CT311 and CT652.1 are likely to interact with nuclear components during infection, and we hypothesize that their nuclear localization enables them to modulate host nuclear processes such as gene expression as other obligate intracellular bacteria secrete effectors to modulate host gene expression (58–62). It will be interesting to determine whether CT652.1 is also localized to the nucleus during infection conditions and whether CT652.1 or CT311 act as nucleomodulins, a bacterial protein that targets the nucleus to modulate nuclear processes (63).

CT620, CT621, CT622, CT631, CT711, and CT712 all localized to the cytoplasm when ectopically expressed. CT620, CT621, CT711, and CT712 contain predicted DUF582 domains. In line with our findings, previous research demonstrated that CT620, CT621, and CT711 are primarily found within the cytoplasm of infected cells and uninfected ectopically expressed cells using immunofluorescence microscopy (34). Additionally, these effectors were also detected in the nuclear fraction of infected host cells via western blotting (34). Of note, our study is the first to successfully evaluate the localization of CT712 in a mammalian cell. Although we did not detect a strong nuclear signal for any of these effectors, immunofluorescence microscopy may not be as sensitive as other detection methods, such as subcellular fractionation. The DUF582 domains of CT620, CT711, and CT712 interact with the membrane remodeling endosomal sorting complexes required for transport-0 (ESCRT-0) protein Hrs in the absence of infection (64). Whether these effectors interact with ESCRT machinery during infection and the functional consequences that arise from this interaction remains to be determined. CT631 was previously evaluated for secretion, but expression was undetected, prohibiting assessment of its secretion. We show here that it is possibly secreted and localizes to the cytoplasm of HeLa cells when ectopically expressed. It will be interesting to determine localization during infection conditions and functional role.

CT142 and CT738 localized to cytoplasmic puncta when expressed in the absence of infection. Notably, CT142 has previously been shown to form globular structures within the inclusion, a phenotype similar to CT143 and CT144 (46). CT738 has been shown to be secreted using surrogate T3SS, but to the best of our knowledge, it has not been evaluated for subcellular localization (36, 52). Since CT142 and CT738 localize to cytoplasmic puncta when expressed in mammalian cells, they may interact with organelles such as vesicles, endosomes, lysosomes, or peroxisomes. These two effectors are particularly intriguing, as *C.t*. infection is known to hijack vesicular trafficking and block lysosomal fusion (65, 66). Additionally, any secreted effector could exhibit altered localization in the context of infection, where interactions with other *C.t*. proteins or post-translation modifications may modulate their trafficking or function.

In conclusion, we evaluated the secretion of 36 putative *C.t*. effectors that were previously evaluated for secretion using a surrogate host. Using a series of three robust secretion assays, we identified 11 proteins that were secreted and an additional 10 proteins that are possibly secreted into the eukaryotic cell. Of these, 10 displayed distinct localization patterns. A few proteins, previously designated as secreted, were not secreted in our assays, underscoring the importance of validating secretion with the native bacterial context. This study provides a valuable resource for the *C.t*. community by identifying candidate effectors for functional characterization and highlighting the limitations of surrogate host approaches. Many of the confirmed effectors remain poorly characterized and represent promising targets for future studies to unravel the molecular mechanisms of chlamydial pathogenesis.

## SUPPLEMENTAL MATERIAL

Table S1 spectrum.01616-25-s0001.docx Primers used in study.

ASM does not own the copyrights to Supplemental Material that may be linked to, or accessed through, an article. The authors have granted ASM a non-exclusive, world-wide license to publish the Supplemental Material files. Please contact the corresponding author directly for reuse.
