# Supplementary material for: Validation and subcellular localization of previously predicted type III secreted effector proteins in Chlamydia trachomatis
Source: Microbiol Spectr. 2025 Oct 24;13(12):e01616-25. doi: 10.1128/spectrum.01616-25 (PMC12671076; doi:10.1128/spectrum.01616-25)
Supplement: Table S1 — Primers used in study. [file spectrum.01616-25-s0001.docx]

Table S1: Primers used for completion of this study. Nucleotides in bold correspond to the restriction site.

| **Primer Name** | **Sequence** | **Uses** |
| --- | --- | --- |
| **Expression in *C.t.*** | | |
| CT016 NotI F | CC**GCGGCCGC**ATGAAAGTCAAAATTAATGATCAGTTC | CyaA, BlaM, GSK assays |
| CT016 KpnI R | CC**GGTACC**AGTATAAAGAACAGCTTTCACGTGTTC | CyaA, BlaM, GSK assays |
| CT053 NotI F | CC**GCGGCCGC**ATGAAAAGTGAGCGTTTAAAAAAATT | CyaA, BlaM, GSK assays |
| CT053 KpnI R | CC**GGTACC**CCATTCATTCGCGTCAGG | CyaA, BlaM, GSK assays |
| CT082 NotI F | CC**GCGGCCGC**ATGTCAATTTCTGGAAGTGGTAATG | CyaA, BlaM, GSK assays |
| CT082 KpnI R | CC**GGTACC**TGAATCGCCGCCTGC | CyaA, BlaM, GSK assays |
| CT083 NotI F | CC**GCGGCCGC**ATGAGAATAATCCCTTTTGATCCTT | CyaA, BlaM, GSK assays |
| CT083 KpnI R | CC**GGTACC**TGAATCGCCGCCTGC | CyaA, BlaM, GSK assays |
| CT142 NotI F | CC**GCGGCCGC**ATGAGTGATTCTGACAAAATTATTAAT | CyaA, BlaM, GSK assays |
| CT142 KpnI R | CC**GGTACC**TCCTCCTATCTCTGGGTATACGAG | CyaA, BlaM, GSK assays |
| CT143 NotI F | CC**GCGGCCGC**ATGAAGAAACCAGTATTTACAGGGG | CyaA, BlaM, GSK assays |
| CT143 KpnI R | CC**GGTACC**ATCTGCCTCCTTATAAGAAGAACCA | CyaA, BlaM, GSK assays |
| CT144 NotI F | CC**GCGGCCGC**ATGACAACACCAGATAATAATACTATTGAT | CyaA, BlaM, GSK assays |
| CT144 KpnI R | CC**GGTACC**AGGAACAACAGGTAGCCGAA | CyaA, BlaM, GSK assays |
| CT161 NotI F | CC**GCGGCCGC**GTGGCTAGAAAACCTTTAGTAGATAGA | CyaA, BlaM, GSK assays |
| CT161 KpnI R | CC**GGTACC**GTCATAAAAATTTTCCATTTCTGTAGG | CyaA, BlaM, GSK assays |
| CT163 NotI F | CC**GCGGCCGC**ATGTTTGTGTCGTTCGATAAATCC | CyaA, BlaM, GSK assays |
| CT163 KpnI R | CC**GGTACC**ATAACGAATGCGACAATACTGC | CyaA, BlaM, GSK assays |
| CT309 NotI F | CC**GCGGCCGC**ATGAATCAGTATTATTTTTTATCCTCCTTC | CyaA, BlaM, GSK assays |
| CT309 KpnI R | CC**GGTACC**CCATCTGATTCCTTTCTCCAT | CyaA, BlaM, GSK assays |
| CT311 NotI F | CC**GCGGCCGC**ATGAAAAGAGTTATCCTCTGCTCTCT | CyaA, BlaM, GSK assays |
| CT311 KpnI R | CC**GGTACC**TTTTCCATTTTGCAGATCTTTCA | CyaA, BlaM, GSK assays |
| CT330 NotI F | CC**GCGGCCGC**ATGCAGGAAATCTCGGTACCT | CyaA, BlaM, GSK assays |
| CT330 KpnI R | CC**GGTACC**TACAGATTCCCCAGGGATAAAAG | CyaA, BlaM, GSK assays |
| CT338 NotI F | CC**GCGGCCGC**ATGGTGTATAGTTATAAAGGAATAGTATATTT | CyaA, BlaM, GSK assays |
| CT338 KpnI R | CC**GGTACC**AGGTTTTTGAGATAAAAGATACT | CyaA, BlaM, GSK assays |
| CT386 NotI F | CC**GCGGCCGC**ATGCAAATTCCAAGAAGTGTTG | CyaA, BlaM, GSK assays |
| CT386 KpnI R | CC**GGTACC**TACTAATCTCTGCTGTTTTAACA | CyaA, BlaM, GSK assays |
| CT429 NotI F | CC**GCGGCCGC**ATGACGACATATCCTGTACCTCAA | CyaA, BlaM, GSK assays |
| CT429 KpnI R | CC**GGTACC**TGAACGGCTCTTCTTACGTCCAC | CyaA, BlaM, GSK assays |
| CT504 NotI F | CC**GCGGCCGC**GTGTATTTTACAAGAGATCCAGTCAT | CyaA, BlaM, GSK assays |
| CT504 KpnI R | CC**GGTACC**CTCTTCTGAAGAAATACTGTC | CyaA, BlaM, GSK assays |
| CT550 NotI F | CC**GCGGCCGC**GTGAGTTTAGATTTTTTAGAGGA | CyaA, BlaM, GSK assays |
| CT550 KpnI R | CC**GGTACC**TCCATCTATTGAAGGCG | CyaA, BlaM, GSK assays |
| CT583 NotI F | CC**GCGGCCGC**ATGGGAAATATTAAAACCCTTTTAGAG | CyaA, BlaM, GSK assays |
| CT583 KpnI R | CC**GGTACC**TCGATTTCTAGAGTTTTGGGTTT | CyaA, BlaM, GSK assays |
| CT606.1 NotI F | CC**GCGGCCGC**TTGGAAGATAGAATGATCGACGG | CyaA, BlaM, GSK assays |
| CT606.1 KpnI R | CC**GGTACC**CTCGCGGGGAAAGAGAGTCT | CyaA, BlaM, GSK assays |
| CT610 NotI F | CC**GCGGCCGC**ATGATGGAGGTGTTTATGAAT | CyaA, BlaM, GSK assays |
| CT610 KpnI R | CC**GGTACC**ATAAGATTGATGACAACTACAAC | CyaA, BlaM, GSK assays |
| CT620 NotI F | CC**GCGGCCGC**ATGTGTTCTATGAACATATTTAATAAAATTAACTC | CyaA, BlaM, GSK assays |
| CT620 KpnI R | CC**GGTACC**ACTAGCCAGTTTTCTTGTTAAACCA | CyaA, BlaM, GSK assays |
| CT621 NotI F | CC**GCGGCCGC**ATGAACCGTATTCATCGTACACAA | CyaA, BlaM, GSK assays |
| CT621 KpnI R | CC**GGTACC**TCTTAAGAGATTACGCGCTAATCC | CyaA, BlaM, GSK assays |
| CT622 NotI F | CC**GCGGCCGC**ATGGAATCAGGACCAGAATCAG | CyaA, BlaM, GSK assays |
| CT622 KpnI R | CC**GGTACC**AGAAAGATAACCAGAGAATAGAGAA | CyaA, BlaM, GSK assays |
| CT631 NotI F | CC**GCGGCCGC**ATGAAAACGTTAATTGATAACA | CyaA, BlaM, GSK assays |
| CT631 KpnI R | CC**GGTACC**TAAACAAATAATTCCTTCAAACT | CyaA, BlaM, GSK assays |
| CT652.1 NotI F | CC**GCGGCCGC**ATGGACCAGTTATCACAGA | CyaA, BlaM, GSK assays |
| CT652.1 KpnI R | CC**GGTACC**ACCTTGGGAATCTTCTT | CyaA, BlaM, GSK assays |
| CT656 NotI F | CC**GCGGCCGC**ATGGACACGCAATTCATAGCG | CyaA, BlaM, GSK assays |
| CT656 KpnI R | CC**GGTACC**ATCTCTGTATACCGAACGCATTT | CyaA, BlaM, GSK assays |
| CT671 NotI F | CC**GCGGCCGC**ATGGAATTAAATAAAACTTCGGAATCT | CyaA, BlaM, GSK assays |
| CT671 KpnI R | CC**GGTACC**TATATGAGCTTCTTCTACTTTCTTCTC | CyaA, BlaM, GSK assays |
| CT711 NotI F | CC**GCGGCCGC**GTGTCAATACAACCTACATCCATTTC | CyaA, BlaM, GSK assays |
| CT711 KpnI R | CC**GGTACC**TTTAAATCTACGGATCAACTTAGCAA | CyaA, BlaM, GSK assays |
| CT712 NotI F | CC**GCGGCCGC**ATGAGAAACCATCCGATTCC | CyaA, BlaM, GSK assays |
| CT712 KpnI R | CC**GGTACC**GCTAGAAGCCAATGTTCTATATACATT | CyaA, BlaM, GSK assays |
| CT718 NotI F | CC**GCGGCCGC**TTGGAGGATTACGTGGCTTC | CyaA, BlaM, GSK assays |
| CT718 KpnI R | CC**GGTACC**TGGAGTGAGTACACTTAGTAGATGGG | CyaA, BlaM, GSK assays |
| CT737 NotI F | CC**GCGGCCGC**ATGACCACTAACTCTACTCAAGACACT | CyaA, BlaM, GSK assays |
| CT737 KpnI R | CC**GGTACC**TTCTTCTTGGGGAACGAAT | CyaA, BlaM, GSK assays |
| CT738 NotI F | CC**GCGGCCGC**ATGGAAGGTTTTTTCCCTATA | CyaA, BlaM, GSK assays |
| CT738 KpnI R | CC**GGTACC**TACAAGGCTCGGAGCAGAAAAGA | CyaA, BlaM, GSK assays |
| CT795 NotI F | CC**GCGGCCGC**ATGAGATTCTTGTTAGCTTTATTCTCA | CyaA, BlaM, GSK assays |
| CT795 KpnI R | CC**GGTACC**CTCAACAAATTCAGGATTTATTG | CyaA, BlaM, GSK assays |
| CT847 NotI F | CC**GCGGCCGC**ATGAGTAGTGCAATCATCCCC | CyaA, BlaM, GSK assays |
| CT847 KpnI R | CC**GGTACC**GTCCGAAGGAGACGTTGTAAG | CyaA, BlaM, GSK assays |
| CT848 NotI F | CC**GCGGCCGC**ATGTGGCATAAAGAACCAATGTATG | CyaA, BlaM, GSK assays |
| CT848 KpnI R | CC**GGTACC**GATATTCGCGATCAAGCTAACG | CyaA, BlaM, GSK assays |
| CT849 NotI F | CC**GCGGCCGC**ATGTCAGCACCAACCTCACA | CyaA, BlaM, GSK assays |
| CT849 KpnI R | CC**GGTACC**AGACAGGGGTTTATTTAATTGGTTAAC | CyaA, BlaM, GSK assays |
| **Ectopic Expression** | | |
| CT016 KpnI F | CC**GGTACC**ATGAAAGTCAAAATTAATGATCAGTTC | Ectopic expression |
| CT016 XhoI R | CC**CTCGAG**AGTATAAAGAACAGCTTTCACGTGTTC | Ectopic expression |
| CT053 KpnI F | CC**GGTACC**ATGAAAAGTGAGCGTTTAA | Ectopic expression |
| CT053 XhoI R | CC**CTCGAG**CCATTCATTCGCGTCAGG | Ectopic expression |
| CT142 KpnI F | CC**GGTACC**ATGAGTGATTCTGACAAAATTATTAAT | Ectopic expression |
| CT142 XbaI R | CC**TCTAGA**TTATCCTCCTATCTCTGGGTATACGAG | Ectopic expression |
| CT143 KpnI F | CC**GGTACC**TTAATCTGCCTCCTTATAAGAAGAACCA | Ectopic expression |
| CT143 XhoI R | CC**CTCGAG**ATCTGCCTCCTTATAAGAAGAACCA | Ectopic expression |
| CT144 KpnI F | CC**GGTACC**ATGACAACACCAGATAATAATACTATTGAT | Ectopic expression |
| CT144 XhoI R | CC**CTCGAG**AGGAACAACAGGTAGCCGAA | Ectopic expression |
| CT161 KpnI F | CC**GGTACC**GTGGCTAGAAAACCTTTAGTAGATAGA | Ectopic expression |
| CT161 XhoI R | CC**CTCGAG**GTCATAAAAATTTTCCATTTCTGTAGG | Ectopic expression |
| CT311 KpnI F | CC**GGTACC**ATGAAAAGAGTTATCCTCTGCTCTCT | Ectopic expression |
| CT311 XhoI R | CC**CTCGAG**TTTTCCATTTTGCAGATCTTTCA | Ectopic expression |
| CT386 KpnI F | CC**GGTACC**ATGCAAATTCCAAGAAGTGTTG | Ectopic expression |
| CT386 XhoI R | CC**GGTACC**TACTAATCTCTGCTGTTTTAACA | Ectopic expression |
| CT504 KpnI F | CC**CTCGAG**GTGTATTTTACAAGAGATCCAGTCAT | Ectopic expression |
| CT504 XhoI R | CC**GGTACC**CTCTTCTGAAGAAATACTGTC | Ectopic expression |
| CT583 KpnI F | CC**GGTACC**ATGGGAAATATTAAAACCCTTTTAGAG | Ectopic expression |
| CT583 EcoRI R | CC**GAATTC**TCGATTTCTAGAGTTTTGGGTTT | Ectopic expression |
| CT620 KpnI F | CC**GGTACC**ATGTGTTCTATGAACATATTTAATAAAATTAACTC | Ectopic expression |
| CT620 XhoI R | CC**CTCGAG**ACTAGCCAGTTTTCTTGTTAAACCA | Ectopic expression |
| CT621 KpnI F | CC**GGTACC**ATGAACCGTATTCATCGTACACAA | Ectopic expression |
| CT621 EcoRI R | CC**GAATTC**TCTTAAGAGATTACGCGCTAATCC | Ectopic expression |
| CT622 KpnI F | CC**GGTACC**ATGGAATCAGGACCAGAATCAG | Ectopic expression |
| CT622 XhoI R | CC**CTCGAG**AGAAAGATAACCAGAGAATAGAGAAGC | Ectopic expression |
| CT631 KpnI F | CCGGTACCATGAAAACGTTAATTGATAACAACATC | Ectopic expression |
| CT631 XhoI R | CC**CTCGAG**TAAACAAATAATTCCTTCAAACT | Ectopic expression |
| CT652.1 KpnI F | CC**GGTACC**ATGGACCAGTTATCACAGATACATCA | Ectopic expression |
| CT651.2 XhoI R | CC**CTCGAGTTA**ACCTTGGGAATCTTCTTCGG | Ectopic expression |
| CT656 KpnI F | CCGGTACCATGGACACGCAATTCATAGC | Ectopic expression |
| CT656 XhoI R | CC**CTCGAGTTA**ATCTCTGTATACCGAACGCATTTT | Ectopic expression |
| CT671 KpnI F | CC**GGTACC**ATGGAATTAAATAAAACTTCGGAATCT | Ectopic expression |
| CT671 XhoI R | CC**CTCGAGTTA**TATATGAGCTTCTTCTACTTTCTTCTC | Ectopic expression |
| CT711 KpnI F | CC**GGTACC**GTGTCAATACAACCTACATCCATTTC | Ectopic expression |
| CT711 XhoI R | CC**CTCGAGTTA**TTTAAATCTACGGATCAACTTAGCAA | Ectopic expression |
| CT712 KpnI F | CC**GGTACC**ATGAGAAACCATCCGATTCC | Ectopic expression |
| CT712 XbaI R | CC**TCTAGA**TTAGCTAGAAGCCAATGTTCTATATACATT | Ectopic expression |
| CT738 KpnI F | CC**GGTACC**ATGGAAGGTTTTTTCCCTATAGC | Ectopic expression |
| CT738 XhoI R | CC**CTCGAG**TACAAGGCTCGGAGCAGAA | Ectopic expression |
| CT848 KpnI F | CC**GGTACC**ATGTGGCATAAAGAACCAATGTATG | Ectopic expression |
| CT848 XhoI R | CC**CTCGAG**TTAGATATTCGCGATCAAGCTAACG | Ectopic expression |
| CT849 KpnI F | CC**GGTACC**ATGTCAGCACCAACCTCACA | Ectopic expression |
| CT849 XhoI R | CC**CTCGAG**TTAAGACAGGGGTTTATTTAATTGGTTAAC | Ectopic expression |
